# Evaluation of the therapeutic effect of dressing containing Silver (Ag coat) in the process of healing skin blisters caused by limb fractures: a clinical trial study

**DOI:** 10.1186/s12893-023-02012-8

**Published:** 2023-04-28

**Authors:** Mehdi Teimouri, Sahar sadat Lalehzar

**Affiliations:** 1 Department of Orthopedic Surgery, School of Medicine, Isfahan University of Medical Sciences, Isfahan, Iran; 2 Department of Orthopedic Surgery, Kashani University Hospital, School of Medicine, Isfahan University of Medical Sciences, Isfahan, Iran

**Keywords:** Blister, Bone fracture, Lower extremity, Silver

## Abstract

**Background:**

The main activity of the skin is to create a protective barrier against damage. Loss of the skin due to injury or disease and failure to regenerate the affected area may result in disability, infection, or even death. We conducted a clinical trial to evaluate the therapeutic effect of dressing containing silver in process of healing skin blisters caused by limb fractures.

**Method:**

This is a pioneering randomized trial that compares the effectiveness of two dressings containing silver (Ag coat) and Gaz Vaseline among patients with skin blisters due to bone fractures who were randomly selected from patients referred to the Kashani Medical Training Center. There were two treatment groups containing 16 patients treated with Ag coat and 15 patients treated with Gaz Vaseline. Pictures were taken of blisters on days 0, 7, and 14 to evaluate the healing process. The amount of pain, duration of the visit (measured by minutes), and general condition of the wound were checked. The amount of pain, duration of visit (measured by minutes) and general condition of the wound was checked. All continuous and categorical data are presented as mean ± standard deviation (SD) and frequency (percentage), respectively. Paired sample T-test and repeated measure analysis of variance (ANOVA), Chi-squared test was used. All pictures were analyzed by Mosaic soft ward.

**Result:**

During this study, there was no significant difference between the mean of age and BMI and frequency of gender in the two study groups (*P* > 0.05). There was a significant difference in mean between the duration of the visit, number of dressings, and net cost of dressing $(p <0.05)$. In the macroscopic study and analysis for evaluation and comparing wound area with the Mosaic soft ward, there was significant relation in time (p1 = 0.00). There is no significant difference between the groups (p2 = 0.84). There was a significant difference between time and group (p3 = 0.00). On day 14 the wound area between groups had a significant difference (p4 = 0.00) (Table 3). In the VAS score there was a significant difference in time, and group (p1,2 = 0.00), there was no significant relation between time and group (p3 = 0.62). On all days the wound area between groups had a significant difference (p4 = 0.00).

**Conclusion:**

In conclusion, Ag coat dressing, not only has a significant effect on wound healing but also, decreases pain, shorter visit time, and its more cost-effective.

## Introduction

Investigating the intricate healing process of wounds is essential to understand the multifaceted nature of the wound environment and its complexity. Preclinical models, such as those used in mice, rabbits, and pigs, can be used to simulate different types of wounds, including acute and impaired ones. To ensure reproducible results during a study, investigators must select appropriate methods for monitoring wound progression. This includes non-invasive protocols such as wound tracing, photographic documentation (including image analysis), biophysical techniques, and/or invasive protocols that require wound biopsies. Wound healing is a complex process involving multiple cell types, cytokines, mediators, and the vascular system. It is the skin's way of protecting the body from environmental damage. Recent research has allowed us to better understand how wound healing works and take effective steps toward better wound care. Studies have shown that immune cell infiltration and messaging play a significant role in scar formation and fibrosis [1, 2]. Mast cells are essential for wound healing, comprising up to 8% of the total number of cells in the dermis. When an injury occurs, mast cells release proinflammatory and immunomodulatory mediators which help clot formation, neoangiogenesis, fibrinogenesis, and reepithelialization. Inhibiting mast cell activation or targeting their mediators may be a way to improve wound healing and reduce inflammation and scarring [3].

Orthopedic surgeons must be aware of the pathophysiology and management options of fracture blisters, as these can significantly affect the outcome for patients. These blisters, which can appear between 6 and 72 h post-injury, can delay definitive fracture treatment and increase the risk for postoperative wound complications. Therefore, it is essential to have a comprehensive understanding of fracture blisters and their management options to achieve a satisfactory result [4].

Open fractures due to severe soft tissue disorders which may lead to devastating complications are considered an orthopedic emergency. Also, closed fractures, especially fractures due to high-energy mechanisms, are often associated with severe soft tissue injury. Researches show that a negative impact on the outcome of patients is because of soft tissue damage, especially skin damage as the first skin defense barrier. In particular,Fracture blisters, develop as a sign of significant tissue damage and appear between 6 to 72 h after injury. They can delay the treatment of definitive fractures for a considerable time and simultaneously increase the risk of postoperative wound complications [4].

In recent years, dressings with different compositions have been introduced to protect and accelerate the healing of skin damage caused by burns, fractures, and other accidents leading to skin damage. Recently, bacterial cellulose (BC) has been used in dressings to reduce the duration of wound healing [5, 6], however, more recent studies have shown that BC alone has no antimicrobial activity [7]. In general, inorganic nanomaterials play an important role in antibacterial applications due to their large area and particle shape properties [8]. Metal nanoparticles and metal oxides, which are well known for their antibacterial properties, include silver (Ag), titanium oxide (TiO2), copper oxide (CuO), and zinc oxide (ZnO). Like other polymeric materials, BC can be used to make composites with metals and metal oxides through various synthetic methods [9]. Numerous studies have shown that BC / Ag composite has antibacterial activity against gram-positive and gram-negative bacteria [10]. This study evaluated bacterial cellulose (BC) impregnated with green synthesized silver nanoparticles (AgNPs) as an effective antimicrobial membrane for wound healing. Tests indicated that the nanoparticles were Ag2O and metallic Ag with an average size of 24-40 nm, and had antibacterial activity against Staphylococcus aureus ATCC 6538 and Pseudomonas aeruginosa ATCC 9027. Fourier transform infrared spectroscopy showed that BC and filter paper could both hold the nanoparticles while scanning electron microscopy showed major distortion effects on bacterial cell morphology. The results suggest BC impregnated with green synthesized silver nanoparticles is highly effective in wound healing treatments [11]. The new research developed a nanocomposite of multi-walled carbon nanotubes (MWCNTs) decorated with silver nanoparticles (Ag NPs). It was created using a wet-impregnation technique followed by thermal treatment in an inert atmosphere. The nanocomposite was tested for antibacterial properties against multiple microbial strains, showing its wide range of use in medical applications and water disinfection [12]. Long-acting silver-based dressings have been supported by recent literature as the superior choice of topical agents for burn wounds, however, more research is needed to provide an evidence-based recommendation on their use. Traditionally, surgeon preference has played a major role in determining the usage of these agents, however, it is essential to evaluate the options using randomized controlled trials to ensure optimal outcomes [13].

Recent studies reported the effect of dressing containing Silver on the healing process of burn wounds, healing at the skin graft site, reducing the risk of infection and hospitalization, and its cost-effectiveness. The present study aimed to investigate the effect of Agicoat silver Nano-Crystalline Dressing dressing, produced by Emad Pharmaceutical Company, on blisters caused by bone fractures.

## Method and material

### Study desgine

This is a clinical trial study conducted according to the guidelines of the Ethical Committee of Isfahan University of Medical Sciences (Ethics code: IR.MUI.MED.REC.1401.025, Iranian Registry of Clinical Trials (IRCT) code: IRCT20221207056744N1). Eligible patients attending the Orthopedic Clinic of Isfahan Research Center, Isfahan, Iran, between 2021, and 2022, were randomly assigned. The date of sending the article to the research vice-chancellor of the Esfahan university of medical science was 15/10/2021, Faculty approval date was 23/02/2022, the date of obtaining the ethics code was 17/04/2022, University approval date was 22/05/2022, and registration of our trial protocol under the scientific mentioned name has been approved in Iranian Registry of Clinical Trials at 08/01/2023. our registration reference is IRCT20221207056744N1.

This is a double-blinded pioneering randomized trial that compares the effectiveness of two dressings containing silver (Ag coat) and Gaz Vaseline among patients with skin blisters due to bone fractures who were randomly selected from patients referred to the Kashani Medical Training Center. All fractures were closed in the lower extremity. Fractures are all simple in both groups. The communitive fractures are excluded at the beginning of the research. After the selection, the participants were randomly assigned to the experimental and control groups using lottery cards, so that the results of their allocation to the groups were accessible only after the completion of the initial evaluations by the research staff. As a single-blinded study Participants were blinded to grouping and assignments.*2.2. Inclusion and exclusion criteria:*

The inclusion criteria included all patients with blisters due to bone fracture, aged between 18–65, and Consent to participate in the study.

The exclusion criteria included Sensitivity to silver, Amputation indication, patients who did not agree to participate in the study, and those who did not participate in the study until the end and did not come for follow-up sessions.

The patients were fully informed about the risks, advantages, and trial process. The informed consent was taken from those patients who met the following requirements. They had been diagnosed after the first admission to the Kashani Hospital. They were aged between 18 and 65 years with non-healing blisters that had been created due to bone fracture. All blisters were located on the leg and foot; finally, the patients read, comprehended, and signed the informed consent specific to the study.

Neither the therapist and analyst nor the patient know about the treatment and control group. A third person who did not know about the research and the type of treatments simply randomized the patients into 2 groups; in group A, they were treated with Agi Coat dressing and in group B, they were treated with Gaz Vaseline for coating the blister. Agi coat dressing, which was used in this study, was produced by Emad Pharmaceutical Company, Razi industrial zone, Isfahan, Iran.

Code and technical specifications of AGICOAT medical dressing pad:

**Table:** 

Iran code:	2,132,121,067,790,001

**Table:** 

GS1	6,260,677,900,322
ISIC	3311
CPC	481

This dressing is a single-layer dressing with a coating of silver nanocrystals, which are coated by the chemical reduction method, silver on a network of nylon fibers with very high flexibility. This layer exerts its antimicrobial and anti-inflammatory effects by slowly releasing silver ions.

The blisters were also divided into Hemorrhagic and non-hemorrhagic groups.

### How to use the dressing

The wound or burn site was cleaned with distilled water, betadine, burn ointment, etc. The dressing was removed from the package and moistened with distilled water. The excess water was taken from the dressing and placed directly on the wound so that the side was placed at least one centimeter outside the wound. Then another absorbent dressing was placed on the back and finally, it was fixed at the wound site with a simple bandage, tape, or glue.

### Evaluation of blisters

**Fig. 1:**
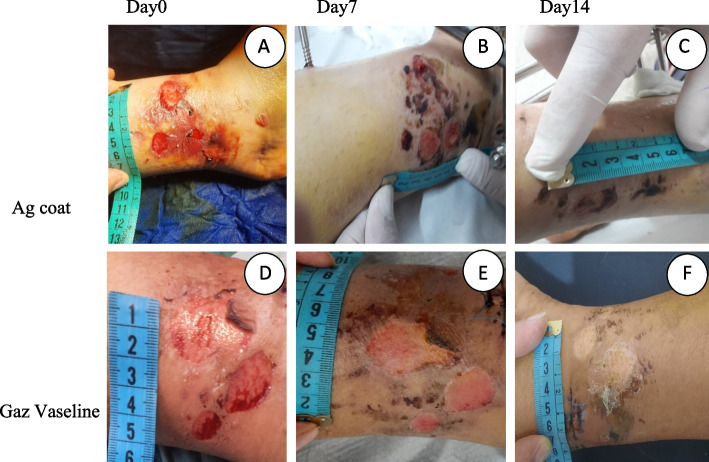
macroscopic view of the blisters and healing process based on dressing type. (**A** & **D**: Blisters in the first day, **B**: day 7 in Ag coat group, **C**: day 14 in Ag coat group, **E**: day 7 in Gaz Vaseline group, **F**: day 14 in Gaz Vaseline group)

The blisters are all superficial) All blisters are drained during the operation (and the width was measured by ruler next to the blister (Fig. 1). Age, gender and BMI were evaluated as a demographic data. The purpose of pain assessment was skin pain during dressing change.and evaluated by Visual Analogue Scale (VAS). Based on this scale, patient’s pain is scored from 0 (least pain) to 10 (highest pain) [14]. Also, Duration of visit(min/day), Duration of visit(min/day), Net cost of dressing (Rial), and compression of hemorragic and non hemorragic blisters, were evaluate.

The blisters were dressed by Agi coat once a week and every day with Gaz Vaseline, and were followed up in day 7 and 14. On all days of hospitalization and clinic visits in both groups, an orthopedic surgeon and a wound specialist visited the patients. The amount of pain, duration of visit (measured by minutes) and general condition of the wound was checked. Photography was done on the first, seventh and fourteenth days after the first day of dressing. Photographs were taken on all days for all patients by one photographic camera, lens perpendicular to the wound and at a distance of fifteen centimeters from the wound. The pictures of wounds were analyzed by a medical doctor with both Mosaic and image j (fiji) soft ward [15].

## Result

From 2020 to 2021, 40 patients with skin blister due to bone fracture entered to study. Data obtained from 31 patients were analyzed after applying the exclusion criteria. The participants were randomly assigned to two. Ag coat groups with 16 participants and Gaz Vaseline group with 15 participants. Table 1 shows the demographic characteristics of the study subjects.

**Table 1:** Determining and comparing the demographic characteristics of the participants according to the study groups

Type of dressing		Agi Coat(16)	Gaz Vaseline(15)	*p*-value
Demographic variables				
Age(mean $\pm$ SD)		40.93 $\pm 6.71$	40.73 $\pm 6.00$	0.93
BMI(mean $\pm$ SD)		24.07 $\pm 0.61$	23.83 $\pm 0.58$	0.28
Gender n(%)	Femail	8(50)	8(46.7)	0.56
	mail	8(50)	7(46.7)	

Table 1 compares the demographic characteristics of the participants according to the study groups. As the results of the Table 1 show, there was no significant difference between the mean of age and BMI and frequency of gender in the two study groups (*P* > 0.05).

In Table 2 we compared duration of visit, number of dressing, net cost of dressing and hemorrhagic wounds between groups. As the results of Table 2 show, there was significant difference mean between duration of visit, number of dressing and net cost of dressing $(p <0.05)$, we expect that healing was considered complete sooner when the blister is non- hemorrhagic, but hemorrhagic and non-hemorrhagic wounds did not have any significant difference between two groups.

**Table 2:** Determining and comparing the frequency of variables

Type of dresssing		Ag coat(16)	Gaz vaseline(15)	*p*-value
variables				
Duration of visit(min/day)		16.68 $\pm 4.19$	27 $\pm 7.02$	0.00
Number of dressings( mean $\pm$ SD)		3 $\pm 0.00$	43.46 $\pm 14.15$	0.00
Net cost of dressing) Rial(		2,425,361.25 $\pm 938118.7564$	8,693,333 $\pm 2830312.115$	0.00
Hemorragic N(%)	Hemorragic	8(50)	9(60)	0.42
	Non-hemorragic	8(50)	6(40)	

In macroscopic study and analysis for evaluation and comparing wound area with the Mosaic soft ward, there were significant relation in time (p1 = 0.00). There is no significant difference between the groups (p2 = 0.84). There was significant difference between time and group (p3 = 0.00). In day 14 the wound area between groups had significant difference (p4 = 0.00) (Table 3). In VAS score there were significant difference in time, group (p1,2 = 0.00), there was no significant relation between time and group (p3 = 0.62). In all days the wound area between groups had significant difference (p4 = 0.00) (Table 3 and Fig. 1).

**Table 3:** Summary of analysis of repeated variance for the average of wound size in treated and control group

		Day0	Week1(day7)	Week2(day14)	P1(time)	P2(group)	P3(time*group)
Wound area(cm^2^)	Agi coat	23.43 $\pm 9.93$	20.65 $\pm 9.61$	17.01 $\pm 9.18$	0.00	0.84	0.00
	Gaz Vaseline	27.28 $\pm 10.89$	26.88 $\pm 10.86$	26.44 $\pm 10.82$			
P4		0.31	0.10	0.01			
Vas Score	Agi coat	5.34 $\pm 0.78$	3.96 $\pm 0.80$	2.56 $\pm 0.60$	0.00	0.00	0.62
	Gaz Vaseline	7.70 $\pm 0.88$	6.50 $\pm 0.96$	5.13 $\pm 0.93$			
P4		0.00	0.00	0.00			

P1, P2 and P3 based on repeated measure ANOVA and P4 based on sample T-test, all variables are presented as mean ± SD

## Discussion

In this study, the patients which their blisters dressed with ag coat dressing had significantly less pain during the time and between groups in compaire with gaz vaselin group, which was mesured by VAS score. Also, in ag coat group the wound healing was significantly faster during the time and in the presence of both groups, which was mesured by analyzed the wound area with the Mosaic soft ward. Duration of visit and the usage of number of dressing was significantly less in ag coat group in compare with gaz vaselin group.

A comprehensive systematic review and meta-analysis shows that the use of nanocrystalline silver dressings reduces the length of hospital stay, reduces pain, requires less surgery, and reduces the rate of infection compared to another kinds of dressing [16]. As the same, in our study also the pain, hospitalization length was less in ag coat group and non of our patients had infection.

In another study, similar to our study, the rate of healing and pain with silver nanocrystal dressing (single-layer dressing) with a coating of silver nanocrystals by chemical reduction method, silver on a network of nylon fibers with the flexibility of this layer with release Slowly silver ions [17] are coated very highly (the results of the study [18] exert their antimicrobial and anti-inflammatory effects) and showed that Ag coat produces faster healing with less pain and is better than the traditional method like gaz vaseline [19].

Two types of burn wound treatment were compared by Mirza Aghazadeh-Attari et al. Silver nanocrystals are a more effective, less costly option for wound healing than silver sulfadiazine ointment. Studies have shown that infection, healing time, pain, need for skin grafts, and hospitalization duration are all lower with silver nanocrystals. Therefore, studies recommended switching to silver nanocrystals for burn wound healing. Exactly similar to our study, this study showed that silver nylon wound dressing reduces the length of hospital stay, analgesia, wound infection, and inflammation compared to another kind of dressing [20].

In the study of Adhya et al., The healing of burn wounds with silver nanocrystals was faster than other dressing, which showed an increase in the healing effect of silver compounds using nanoparticles [21]. Some studies have also shown that compounds. Silver nanocrystals with biocompatible nanofibers can provide faster healing. Liu et al. Reported that the presence of silver nanocrystals increased granulation, increased epithelialization, increased keratinocyte activity, and reduced inflammation, resulting in better wound healing. In addition, the antimicrobial properties of silver make it advisable for any wound, provided It is affordable in terms of price [22].

The mentioned studies and other studies in this field have examined the antimicrobial properties of Agicoat silver Nano-Crystalline Dressing due to its properties such as induction of tissue granulation, increased epithelization, increased activity of keratinocytes, reduced inflammation, and reduced pain, and in order to its cost-effectiveness, it is recommended for any casualty [23].

## Conclusion

One of our limitations in this study is the small sample size. Because most of the patients didn’t come for the follow-up visits on the seventh and fourteenth day of the study, we couldn’t evaluate the exact changes in Wound healing. The estimated duration of the project was 2 to 3 months, but due to the problems raised, the accurate collection of samples took 6 months. In conclusion, the new Ag coat dressing which we used in this study has a significant effect on wound healing and cell proliferation, decreased pain, low hospitalization time, and more cast benefits according to the number of dressings during the treatment period and Speed of wound healing.

## Data Availability

The datasets generated and/or analyzed during the current study are not publicly available due to the nature of patients with humeral shaft fracture but are available from the corresponding author on reasonable request.

## References

[1] Takeo M, Lee W, Ito M. Wound healing and skin regeneration. Cold Spring Harb Perspect Med. 2015;5(1):a023267. 10.1101/cshperspect.a023267 PMC4292081 25561722

[2] Masson-Meyers DS, Andrade TA, Caetano GF, Guimaraes FR, Leite MN, Leite SN, Frade MA (2020). Experimental models and methods for cutaneous wound healing assessment. Int J Exp Pathol.

[3] Komi DE, Khomtchouk K, Santa Maria PL (2020). A review of the contribution of mast cells in wound healing: involved molecular and cellular mechanisms. Clin Rev Allergy Immunol.

[4] Tosounidis TH, Daskalakis II, Giannoudis PV (2020). Fracture blisters: pathophysiology and management. Injury.

[5] Shahbazi F (2019). Analysis of mortality rate of road traffic accidents and its trend in 11 years in Iran. Arch Trauma Res.

[6] Sulaeva I (2015). Bacterial cellulose as a material for wound treatment: Properties and modifications A review. Biotechnol Adv.

[7] Zhu W (2015). In-situ biopreparation of biocompatible bacterial cellulose/graphene oxide composites pellets. Appl Surf Sci.

[8] Moghimi SM, Farhangrazi ZS (2014). Just so stories: the random acts of anti-cancer nanomedicine performance. Nanomedicine.

[9] Beyth N, et al. Alternative antimicrobial approach: nano-antimicrobial materials. Evid-Based Complement Altern Med. 2015;2015. 10.1155/2015/246012 PMC4378595 25861355

[10] Wu J, Zheng Y, Song W, Luan J, Wen X, Wu Z, Chen X, Wang Q, Guo S. In situ synthesis of silver nanoparticles/bacterial cellulose composites for slow-released antimicrobial wound dressing. Carbohyd Polym. 2014;102:762–71. 10.1016/j.carbpol.2013.10.093 24507345

[11] Shaaban MT, Zayed M, Salama HS (2023). Antibacterial Potential of Bacterial Cellulose Impregnated with Green Synthesized Silver Nanoparticle Against S. aureus and P. aeruginosa. Curr Microbiol..

[12] Hamouda HI, Abdel-Ghafar HM, Mahmoud MH (2021). Multi-walled carbon nanotubes decorated with silver nanoparticles for antimicrobial applications. J Environ Chem Eng.

[13] Hashmi DL, Haith L (2019). The current state of topical burn treatments: a review. Current Trauma Reports.

[14] Heller GZ, Manuguerra M, Chow R (2016). How to analyze the Visual Analogue Scale: Myths, truths and clinical relevance. Scand J Pain.

[15] Shivanandan A, Radenovic A, Sbalzarini IF (2013). MosaicIA: an ImageJ/Fiji plugin for spatial pattern and interaction analysis. BMC Bioinformatics.

[16] Lairet KF (2014). Evaluation of an oxygen-diffusion dressing for accelerated healing of donor-site wounds. J Burn Care Res.

[17] Sivakumar AS, Krishnaraj C, Sheet S, Rampa DR, Kang DR, Belal SA, Kumar A, Hwang IH, Yun SI, Lee YS, Shim KS. Interaction of silver and gold nanoparticles in mammalian cancer: as real topical bullet for wound healing—A comparative study. In Vitro Cellular & Developmental Biology-Animal. 2017;53:632–45. 10.1007/s11626-017-0150-5 28462492

[18] Adomavičiūtė E, et al. Formation and biopharmaceutical characterization of electrospun PVP mats with propolis and silver nanoparticles for fast releasing wound dressing. BioMed Res Int. 2016;2016. 10.1155/2016/4648287 PMC4769747 26981531

[19] Momeni M, Fatemi MJ, Kamranfar B, Saberi M, Bagheri T, Niazi M (2019). Comparison of the three dressing methods on the speed of repair and remaining scar on partial-thickness skin graft donor sites in burn patients. Tehran Univ Med J.

[20] Mirza Aghazadeh-Attari A, Lotfi M, Arzani A, Doshmangir L (2021). A comparison of cost-effectiveness of silver nanocrystal dressing with silver sulfadiazine in burn wound healing: a systematic review. J Isfahan Med School.

[21] Adhya A (2014). Healing of burn wounds by topical treatment: A randomized controlled comparison between silver sulfadiazine and nano-crystalline silver. J Basic Clin Pharm.

[22] Liu M, Luo G, Wang Y, Xu R, Wang Y, He W, Tan J, Xing M, Wu J. Nano-silver-decorated microfibrous eggshell membrane: processing, cytotoxicity assessment and optimization, antibacterial activity and wound healing. Scientific reports. 2017;7(1):1–4. 10.1038/s41598-017-00594-x PMC5428678 28348388

[23] Vejdan SA, Khosravi M, Zojaji F. Burn donor site dressing using melolin and flexigrid versus conventional dressing. Shiraz E-Med J. 2015;16(1).

